# Do agreements between adolescent and parent reports on family socioeconomic status vary with household financial stress?

**DOI:** 10.1186/1471-2288-11-50

**Published:** 2011-04-19

**Authors:** Christy Pu, Nicole Huang, Yiing-Jenq Chou

**Affiliations:** 1 Institute of Hospital and Health Care Administration, School of Medicine, National Yang-Ming University, Taipei, Taiwan; 2 Department of Public Health, School of Medicine, National Yang-Ming University, Taipei, Taiwan

## Abstract

**Background:**

Many studies compared the degree of concordance between adolescents' and parents' reports on family socioeconomic status (SES). However, none of these studies analyzed whether the degree of concordance varies by different levels of household financial stress. This research examines whether the degree of concordance between adolescents' and parent reports for the three traditional SES measures (parental education, parental occupation and household income) varied with parent-reported household financial stress and relative standard of living.

**Methods:**

2,593 adolescents with a mean age of 13 years, and one of their corresponding parents from the Taiwan Longitudinal Youth Project conducted in 2000 were analyzed. Consistency of adolescents' and parents' reports on parental educational attainment, parental occupation and household income were examined by parent-reported household financial stress and relative standard of living.

**Results:**

Parent-reported SES variables are closely associated with family financial stress. For all levels of household financial stress, the degree of concordance between adolescent's and parent's reports are highest for parental education (κ ranging from 0.87 to 0.71) followed by parental occupation (κ ranging from 0.50 to 0.34) and household income (κ ranging from 0.43 to 0.31). Concordance for father's education and parental occupation decreases with higher parent-reported financial stress. This phenomenon was less significant for parent-reported relative standard of living.

**Conclusions:**

Though the agreement between adolescents' and parents' reports on the three SES measures is generally judged to be good in most cases, using adolescents reports for family SES may still be biased if analysis is not stratified by family financial stress.

## Background

Socioeconomic status (SES) is an important component in studies that investigates health disparities for adolescents, as SES is associated with many health behaviors as well as health outcomes for adolescents [1-5]. The validity of the results from these studies depend on the accuracy of these SES measures. Previous studies that compare concordance between adolescent and parent-reported SES however, have ignored whether such concordances are in fact affected by household financial stress [6-9].

Household income, parents' educational attainment and occupation class are most commonly used to measure SES for adolescents, and it has been argued that adolescents are unable to report these variables accurately. Wardle et al.[10] found in a sample of 1,824 adolescents that was comprised mainly of girls, approximately 16% of the subjects either did not or could not determine their parents' occupation, and approximately 25% of the subject could not answer whether their parents have a college degree. Similarly, Currie et al.[11] in a sample of 4,079 Scottish schoolchildren found that over 20% of 11-15 year olds were unable to provide a substantive response on father's occupation. However, for studies that compare adolescent's own report on parental SES and that reported by the parents, most found moderate to good agreement depending on the SES measure in question. A study[6] used a Spanish sample of adolescents (n = 91) and found that percentage of agreement for parental education and occupation ranged from 63.6 ~ 87.7% (kappa statistic ranged from 0.39 ~ 0.77). In another study of Norwegian sample (n = 924), agreement of reports for parental occupation was judged to be acceptable (kappa ranging from 0.65 ~ 0.86). Similar results were found by Vereecken and Vandegehuchte[7](Flanders sample, n = 235).

However, it should be noted that degree of concordance between adolescent and parent reports on the SES variables may be different for households experiencing different levels of financial stress. Financial stress has been defined by most of the previous studies has whether the household has enough money to pay for its expenses or have money left over during a period of time before the survey. Respondents are normally asked to choose from a Likert scale response (for example, from *more than enough *to *very short*, or from *a lot of difficulties *to *no difficulties*, or from *a lot of money left over *to *shortage*)[12-15]. Household financial stress is important to look at because it reflects material availability and if concordance varies by actual economic resource of the household, then using proxy measures on these SES variables could lead to biases depending on the level of financial stress the household experiences.

Studies have shown that family financial stress plays important roles in adolescent's health and development [12,13]. Family financial stress has been shown to be associated with depressive symptoms, delinquency and drug use for adolescents [14,16]. A study indicated that family financial stress has adverse effect on adolescents' self-rated health[17]. In addition, family financial stress can lead to suicidal ideation for adolescents through its impact on parent's behavior (such as parental hostile behavior and physical abuse)[18]. It is unreasonable to assume that the degree of concordance for the SES measures is uniformly distributed by family financial stress, and if concordances of the SES measures vary by family financial stress, then estimates of SES disparities in adolescent health using traditional SES measures could be biased even in cases where high concordance between adolescent and parent's reports are found.

Studies have shown that while education, occupation and income have been widely used to measure SES, these three variables may behave very differently from each other in determining health behaviors and health outcomes[19-21]. Specifically, these three variables represent different aspects of social class[8]. In this research, we use all three measures of family SES to determine whether the degree of concordance vary by family financial stress.

## Methods

### Data

The Youth Project was a panel survey conducted by the Academia Sinica of Taiwan in 2000, which is a publicly released dataset. A multi-stage stratified sampling method was used for this study. The various criteria used for the sampling including urbanization degree, proportion of student population in each stratum, and mean student number of the class in each stratum. A total of 29 sub-regions were obtained. Classes were chosen at random within each stratum. Once a class is chosen, all students within the class as well as their parents were interviewed. Overall, 81 classes were chosen from 40 schools (16 from Taipei City, 15 from Taipei County and 9 from Yi-Lan County). The details of the survey design can be found elsewhere[22,23]. The survey consists of 2690 students who were in their first year of junior high school (mean age = 13 years). Of the 2,690 adolescents, 27 of them did not have a matching parent questionnaire. For the remaining 2,663 adolescents, eight had a parent questionnaire returned but it was indicated by the adolescents that the questionnaires had been filled out by themselves, and thus were classified as without a matching parent questionnaire. Of the remaining 2,655 subjects, eight parent questionnaires indicated that the respondent was a grandparent and 37 indicated that the respondent was in "another" relationship with the child and 17 had missing values for the relationship question. Since the student questionnaire only asked for the mother and father's educational attainment and occupation, in order to be conservative, these 45 subjects were not included in the analysis for adolescent-parent agreement. This means that a total of 2,593 adolescents with a matching parent questionnaire that was filled out by the parent (either father or mother) were available for this study.

### SES measures

The adolescents were asked the question "What is your father's occupation?", and in a separate question "What is your mother's occupation?". Both of these questions are open-ended and were coded based on standards developed by the Youth Project. Eventually the occupations were classified into (1) Managerial; (2) Professional; (3) Highly skilled; (4) Semi-skilled; (5) Agricultural workers; (6) Low skilled; (7) Unskilled; (8) Military; (9) Unemployed/not in job, and (10) Unable to classify. Category (9) included those who were unemployed, housewives, students, and those with no-wage jobs. Class (10) includes answers that only indicated the type of industry and were without a job title.

In the parent questionnaire, the questions "What is your occupation?" and "What is your spouse's occupation?" were asked. Both are open ended questions with a similar coding method to that mentioned above. The questionnaire also asked the responding parent to determine the relationship with the child, and the parent was able to choose from "Mother", "Father", "Grandparent" and "Other". The first two relationships (mother or father filling out the questionnaire) represented 97.3% of the entire sample.

The adolescents were asked to report the monthly household income, and the answer used a 12-category scale ranging from "Below NT$30,000" (approximately US$1,000, based on the exchange rate NT$30 = US$1) to "Above NT$150,000" (approximately US$5,000). The parent was asked to report the monthly household income by an open ended question "The monthly household income of your family is approximately ___thousands of NT$". The answer from the parent's report on this question was then coded into the same interval as the one from the adolescent questionnaire described above.

Finally, the adolescents were asked to report their father and mother's educational attainment using two separate questions, and the parents were asked to report their own as well as their spouse's educational attainment level. This variable consisted of six categories ranging from "primary school or below" to "graduate".

### Financial stress

To see whether the degree of concordance vary by household financial stress, we included analyses using two parental perceived financial stress questions. The parents were asked the question "How has the financial status of your household been for the past year before the interview?" and the respondent can choose from "a lot surplus", "some surplus", "balanced" and "shortage". Asking for financial balances is a typical approach to measuring financial stress and has been used in many studies [15]. To measure *relative *financial stress, the parents were asked in a separate question "How is the current standard of living of your household compared with other households?" and the respondent can choose from "a lot better", "better", average", "worse" and "a lot worse".

### Statistical analysis

Descriptive statistics were used to show distribution of the responses by adolescents and parents. We first compared the distribution of responses, including those with missing responses, for the three SES variables, namely mother and father's occupation, mother and father's educational attainment (Table 1) and household income (Table 2) for those with a matching parent questionnaire. The degree of agreement was analyzed using the weighted κ. Cohen's κ has been widely used to compare answers for similar questions by different respondents [24-26]. For ordinal variables, weighted κ should always be used so that partial credits are given to responses in the adjacent group but not in exact concordance[27]. Following Whiteman and Green[28], we weighted the κ statistic by the square of the deviation from exact agreement. Such weights were applied to parental education and household income, since these two measures of SES have a clear ordered rank. No weight was applied to parental occupation since it is a multinomial variable. Guidelines for assessing the degree of agreement have been proposed as follows[8]: 0.8 < κ ≦ 1 (very good), 0.6 < κ ≦ 0.8 (good), 0.4 < κ ≦ 0.6 (moderate), 0.2 < κ ≦ 0.4 (fair) and κ ≦ 0.2 (poor).

**Table 1:** Adolescent and parent's responses for parental SES.

n = 2,593	Father's SES		Kappa (95%CI)	Mother's SES		Kappa (95%CI)
	Adolescent	Parent		Adolescent	Parent	
**Parent's education**						
*Primary school or below*	13.7	15.0	0.86 (0.85 - 0.88)	17.5	19.2	0.84 (0.82 - 0.86)
*Junior high school*	25.6	27.0		25.6	27.3	
*Senior high school*	24.7	15.3		25.6	12.3	
*3-year technical school*	7.8	14.2		9.9	22.7	
*5-year technical school*	6.5	11.1		5.4	7.6	
*University*	10.8	10.0		7.9	6.1	
*Graduate*	3.5	2.5		1.3	0.9	
*Non response*	7.4	4.9		6.7	3.9	
**Parent's occupation**						
*Managerial*	12.6	14.1	0.39 (0.36 - 0.41)	4.4	7.5	0.47(0.45 - 0.49)
*Professional*	5.9	8.2		4.9	5.2	
*Skilled*	10.3	7.9		7.9	8.3	
*Semi-skilled*	23.5	25.8		26.5	19.4	
*Agricultural*	3.3	3.4		0.5	0.2	
*Low skilled*	26.1	25.3		10.9	9.1	
*Unskilled*	8.1	3.7		6.7	2.2	
*Military*	0.8	5.6		0.1	0.1	
*Unemployed/not in job*	2.5	1.3		33.9	37.3	
*Unable to classify*	0.9	2.7		0.4	3.6	
*Non response*	6.0	10.3		3.9	12.5	

**Table 2:** Adolescent and parent's responses for household income.

n = 2,593	Adolescent	Parent	Kappa(95%CI)
<NT$30,000	16.1	10.2	0.44(0.39 - 0.49)
NT$30,000-49,999	20.2	17.6	
NT$50,000-59,999	19.3	12.2	
NT$60,000-69,999	6.4	7.2	
NT$70,000-79,999	7.6	4.4	
NT$80,000-89,999	4.2	6.1	
NT$90,000-99,999	4.2	2.2	
NT$100,000-109,999	3.4	6.5	
NT$110,000-119,999	2.8	0.5	
NT$120,000-129,999	1.5	1.0	
NT$130,000-139,999	1.0	0.4	
NT$140,000-149,999	0.6	0.3	
>NT$150,000	3.6	3.3	
Non response	9.2	28.2	

US$1 = NT$30

The distributions of the parent-reported SES variables by levels of household financial stress are presented in Tables 3, 4, 5. We then compare the degree of concordance between adolescents' report and the parents' reports relative to the two parent-rated household financial stress questions (Table 6). It should be note that if both parties (adolescent and parent) had non-response, such non-responses may also be considered "consistent" between the two parties. Previous studies usually ignore cases with missing responses in calculating Cohen's κ. However it should be noted that missing responses may not be at random, and it is most likely that those belong to the lower SES groups are actually the ones who are more likely to have missing responses. Thus as a sensitivity analysis, we tested whether results changed by treating pairs of respondent (adolescent and parents) as having consistent responses if both parties had non-responses. To do so, we generated a separate group for the three SES measures for cases where both the parents and the adolescent had missing responses. For parental education and household income, this new group is ranked as the lowest (since it is reasonable to assume that it is more likely for the low SES households to have missing values for both parties) and weights still applied in calculating the κ statistic. For parental occupation, this new group is treated as an independent group in the multinomial variables for occupation. 95% confidence intervals (95%CI) were calculated using the bootstrapping method. A significant difference of κ is determined by non-overlapping 95% CI.

**Table 3:** Distribution of parent-reported education by financial stress (%)

	Father's education				Mother's education			
	Surplus	Balanced	Shortage		Surplus	Balanced	Shortage	
n	874	1287	403	*p*	874	1287	403	*p*
Primary school or below	8.4	16.4	23.6	**	11.0	22.3	26.3	**
Junior high school	17.5	32.3	30.5		19.2	30.8	33.5	
Senior high school	14.3	16.6	14.4		11.4	13.0	12.2	
3-year technical school	16.5	13.2	12.2		27.6	20.5	19.1	
5-year technical school	16.5	9.3	5.5		13.6	5.1	3.2	
University	18.5	6.6	3.2		12.5	3.0	2.7	
Graduate	5.4	0.9	1.5		2.1	0.4	0.2	
Non-response	3.0	4.6	9.2		2.6	4.9	2.7	

**χ^2 ^test for difference in distribution by levels of financial stress, p < 0.000

**Table 4:** Distribution of parent-reported parental occupation by financial strain (%).

	Father's occupation				Mother's occupation			
	Surplus Balanced Shortage				Surplus Balanced Shortage			
	874	1287	403	*p*	874	1287	403	*p*
Managerial	10.5	3.7	2.7	**	3.7	1.1	1.5	**
Professional	11.7	6.4	6.7		7.1	4.5	3.2	
Skilled	12.2	6.4	4.0		11.6	6.6	7.0	
Semi-skilled	29.8	25.3	18.6		23.2	17.7	16.6	
Agricultural	2.3	3.6	5.0		0.1	0.2	0.0	
Low skilled	17.5	28.4	32.3		4.2	11.2	13.2	
Unskilled	2.2	4.4	5.0		1.1	2.3	4.5	
Military	5.5	6.2	3.7		0.1	0.2	0.0	
Unemployed/not in job	0.5	1.2	3.2		34.6	39.2	37.7	
Unable to classify	2.1	2.9	3.2		3.9	3.3	4.2	

**χ^2 ^test for difference in distribution by levels of financial stress, p < 0.000 US$1 = NT$30

**Table 5:** Distribution of parent-reported household income by financial stress (%)

	Surplus	Balanced	Shortage	
n	874	1287	403	*p*
<NT$30,000	4.92	11.19	17.87	**
NT$30,000-49,999	11.78	19.89	22.83	
NT$50,000-59,999	9.95	14.14	10.67	
NT$60,000-69,999	8.92	7.3	3.72	
NT$70,000-79,999	6.29	3.73	2.23	
NT$80,000-89,999	9.04	5.13	3.23	
NT$90,000-99,999	3.89	1.55	0.5	
NT$100,000-109,999	12.36	3.73	2.7	
NT$110,000-119,999	1.03	0.31	0.0	
NT$120,000-129,999	1.95	0.54	0.3	
NT$130,000-139,999	0.92	0.16	0.3	
NT$140,000-149,999	0.69	0.16	0.0	
>NT$150,000	7.44	1.17	1.2	
Non response	20.8	31.0	34.5	

**χ^2 ^test for difference in distribution by levels of financial stress, p < 0.000

**Table 6:** Kappa statistics for concordance for adolescent and parent reports of SES, by parent reported financial condition.

	n	Father education		Mother education		Father occupation		Mother occupation		Household income	
Parent reported financial status		κ	95% CI	κ	95% CI	κ	95% CI	κ	95% CI	κ	95% CI
*Surplus*	874	0.87	(0.85 - 0.90)	0.83	(0.76 - 0.87)	0.42	(0.38 - 0.47)	0.50	(0.46 - 0.55)	0.43	(0.35 - 0.50)
*Balanced*	1,287	0.85	(0.82 - 0.87)	0.78	(0.75 - 0.83)	0.34	(0.30 - 0.37)	0.46	(0.42 - 0.50)	0.34	(027 - 0.42)
*Not enough*	403	0.78	(0.66 - 0.84)	0.71	(0.59 - 0.80)	0.39	(0.31 - 0.44)	0.40	(0.35 - 0.45)	0.31	(0.19 - 0.42)
Parent reported SOL											
*A lot better/better*	313	0.87	(0.82 - 0.90)	0.86	(0.80 - 0.91)	0.39	(0.32 - 0.46)	0.49	(0.44 - 0.57)	0.39	(0.28 - 0.54)
*Average*	1,952	0.86	(0.83 - 0.87)	0.81	(0.77 - 0.84)	0.38	(0.36 - 0.41)	0.47	(0.44 - 0.50)	0.39	(0.33 - 0.45)
*Worse/a lot worse*	303	0.84	(0.70 - 0.89)	0.66	(0.51 - 0.83)	0.32	(0.26 - 0.39)	0.40	(0.34 - 0.47)	0.36	(0.19 - 0.54)

Non-response = 29 for financial strain and 25 for standard of living.

## Results

The distributions of responses by adolescents and parents for parental education, parental occupation and household income are presented in Tables 1 and 2. Adolescents have higher non-response rate for parental education compared with parent's responses. However, for parental occupation and family income, adolescents actually had lower non-response rate than their parents.

For both adolescents and parents, the highest non-response rate was observed for family income, compared with other SES measures. The κ statistic showed good agreements between the reports by adolescents and by parents for both father and mother's educational attainment levels (κ = 0.86, 95% CI = 0.85-0.88 and κ = 0.84, 95% CI = 0.82-0.86, respectively). The degree of concordance is lower for parental occupation (κ = 0.39, 95% CI = 0.36-0.41 and κ = 0.47, 95% CI = 0.45-0.49, for father's and mother's occupation respectively). For household income, κ = 0.44 (95% CI = 0.39-0.49).

The relationship between parent-reported SES and financial stress is shown in Table 3, 4, 5. The results showed that household financial stress is closely associated with the SES variables. For example, out of the parents who reported "surplus" for the financial stress question, 23.9% of them reported father's education equal or above university (Table 3). The corresponding figures for those parents who reported "average" and "shortage" were only 7.5% and 4.7% respectively. Similar patterns were observed for maternal education and household income. For parental occupation, a much higher percentage of the parents who reported "surplus" belong to the occupation categories of managerial, profession and skilled occupations.

The degree of concordance for the three SES measures relative to financial stress and relative standard of living from the parent's perception is reported in Table 6. For parental education and occupation, the degree of concordance is the lowest for parents reported highest financial stress. For father's education for example, κ decreased from 0.87 (95% CI = 0.85-0.90) for families with *surplus *in the financial stress question to 0.78 (95%CI = 0.66-0.84) for those with worst financial balances. The non-overlapping 95% CI indicates that the decrease in κ is statistically significant. Similar pattern was observed for father's occupation (between those who reported *surplus *and *balanced*), and mother's occupation (between those who reported *surplus *and *not enough*). Such pattern of decreasing concordance was less significant for the *relative standard of living *question.

As a sensitivity analysis, we also tested whether the results remain if treating concordance for missing values as a separate group (Tables not shown). The results from this analysis were similar to the ones without missing values.

## Discussions

We found that the degree of concordance for the three SES measures between adolescent and parent reports decreases with higher family financial stress, and the results hold if treating both parties having missing values has concordant.

The role financial stress plays in relation to the traditional SES variables should be discussed. Some suggested that financial stress reflects resources availability, which may not necessarily be captured by the tradition SES measures, as households with similar incomes can show a wide variation in financial stress[15] In addition, financial stress may act as an intermediate factor between the SES and health relationship[29]. However, our data indicated that financial stress is highly correlated with the SES variables in this research, where lower financial stresses were observed for parents with higher education, more skilled jobs and higher household income. This indicates that financial stress may reflect to a large extent the "true" SES of the households. The implication of our results thus is significant: the concordance between the parent and adolescent reported SES variables may be actually determined by the "true" underlying SES of the household. Thus the bias would be larger for higher financial stressed families when estimating the SES gradient for adolescent health. Such bias could arise regardless of whether the SES variables were reported by the parents or by the adolescents.

There are some possible mechanisms for the results found. First, it is possible that adolescents from families with higher financial stress are less aware of the SES situations of the family. For example, it is possible that poor parent-adolescent communication is more prevalent among higher financially stressed families, and hence adolescents were not told about the situations associated with family SES. Second, it is also possible that parents or adolescents from higher financially stressed families are more reluctant to report correct SES data. Previous studies have reported that SES is closely related to self-esteem for adolescents [30], showing that adolescents may be sensitive to their family SES conditions and hence may be reluctant to report accurate SES data when SES is low (which could be reflected by higher family financial stress). These mechanisms can be tested by future studies.

We also found that the phenomenon of decrease in concordance was not observed when using *relative standard of living*. This may indicate that the above proposed mechanisms work more when the household experience *actual *financial difficulties. This may also explained by the previous findings that people may use different reference group to rate their relative standings, and the ratings are sensitive to different reference groups used[31]. In other words, a parent may report *better *in the relative standard of living question while another parent with similar SES may report *worse*, causing the relative standard of living question being less associated with a household's SES situation.

Our results show that concordance for household income is not affected by household financial stress. One obvious explanation is that these two variables are highly related. In other words, parents report their level of financial stress based on household income to a large extent, thus causing less variation of concordance for income when stratified by financial stress.

There are several strengths and weaknesses of this research. In terms of strength, first, though household financial stress and relative standard living may be closely correlated with the three SES measures, the formal two suffer from much less non-responses. The non-response rates for the financial stress variables were only about 1% of the sample. Thus this allows us to test whether concordances of the SES variables are actually affected by a proxy of the "true" SES. Using financial stress has the additional advantage of incorporating factors that are normally not considered when measuring SES (such as whether family members include those with illness, which would reduce available household resources), and hence may be able to better reflect the actual SES condition of the household.

The weakness of this research should also be noted. Like all self-assessed variables, there could be measurement error. For example, a parent may report the household has surplus of income when in fact it does not. For the SES measures, it should also be noted that it is possible that reports by adolescents and parents for the SES measures are both incorrect and hence even a high agreement does not indicate a valid answer. This however, is beyond the scope of this study. In addition, Lien et al.[8] inferred from another study that if both the parent and the children do not report accurately on a particular question, it is more like to be a problem in the response from the family rather than a problem of invalidity caused by the adolescent's lack of understanding of the family situation. Another limitation is that the data used in this study are not very recent. It is possible that individual or societal factors that may influence concordance changed over the past years. It should also be noted that the degree of agreement may not be stable across time, depending on factors such as employment status and feelings of economic security. A solution would be to conduct a test-retest analysis within a period of time. This however, is not available from our data. Finally, the sample used here is representative of Northern Taiwan, and not the entire country. However, Northern Taiwan is of higher SES compared with other parts of the island, and it is reasonable to hypothesize that concordance is higher for the higher SES families. Thus the degree of poor concordance may be underestimated (instead of overestimated).

Several studies found moderate to good concordance between adolescents and parents reports on SES variables such as parental education, parental occupation and household income [6-8,32,33]. Other than the traditional SES measures, a study found good concordance between these two parties on receipt of public assistance[33]. However, whether such concordance influence by household financial stress should be considered. Our results also suggests that in the presence of different levels of financial stress, parental education may still be the most consistent measure of adolescent SES, since its concordance remained higher than other SES variables for all levels of financial stress.

Due to the generally high number of missing values for traditional parental SES variables reported by adolescents, many studies have used the "family affluence scale" (FAS), which included car ownership, telephone ownership and the child having their own unshared bedroom[11,26]. The advantage of using such scale is a lower level of missing data. While a adolescent may not know their parent's educational attainment, they are very likely to know whether their family owns a car[10]. It has also been found that the FAS is highly correlated with a country's gross domestic product[26]. Limitations of the FAS have also been proposed. A study argued that results from FAS are generally non-comparable due to variations in cultures and differences in time[8]. For example, the possibility of owning a car may become much higher for now compares with a decade ago. Some also argued that complete measures of SES should cover both resource-based and prestige-based measures[34]. For these reasons, traditional measures of SES should not be replaced by wealth[35]. This research does not intend to discuss the validity of FAS and whether traditional SES measures should be replaced by FAS in measuring SES for adolescents. However, our results point to some new directions for future research in this area. Firstly, there is a need to consider whether consistency between the adolescent's and parent's report on FAS also decreases with financial stress, and whether the results vary by culture and time. Secondly, it is important to determine the mechanisms for the relationship found between the traditional SES measures and household financial stress and standard of living. By doing this, while it will still be difficult to know the true values for the missing data by either the adolescent or their parent, at least one can postulate a possible upward or downward bias in the estimated SES gradients for adolescents.

## Conclusions

Though the agreement between adolescents' and parents' reports on parental education, parental occupation and household income is generally judged to be good by previous research, using adolescents reports for family SES may still be biased if analysis is not stratified by family financial stress, as the degree of concordance for the three SES measures between adolescent and parent reports decreases with higher family financial stress.

## Competing interests

The authors declare that they have no competing interests.

## Authors' contributions

CP was responsible for data analysis and writing up the manuscript; NH designed the study and helped in drafting the manuscript. YC coordinated the research and finalized the manuscript. All authors read and approved the final manuscript.

## Pre-publication history

The pre-publication history for this paper can be accessed here:

http://www.biomedcentral.com/1471-2288/11/50/prepub
